# Tumor-associated neutrophils induce EMT by IL-17a to promote migration and invasion in gastric cancer cells

**DOI:** 10.1186/s13046-018-1003-0

**Published:** 2019-01-07

**Authors:** Sen Li, Xiliang Cong, Hongyu Gao, Xiuwen Lan, Zhiguo Li, Wenpeng Wang, Shubin Song, Yimin Wang, Chunfeng Li, Hongfeng Zhang, Yingwei Xue, Yuzhou Zhao

**Affiliations:** 1 Department of General Surgery, The Affiliated Cancer Hospital of Zhengzhou University, 127 Dong Ming Road, Zhengzhou, 450008 China; 2 Department of Gastroenterological Surgery, Harbin Medical University Cancer Hospital, 150 Ha Ping Road, Harbin, 150081 China; 3 Department of Gynecologic Oncology, Cancer Hospital Chinese Academy of Medical Sciences & Peking Union Medical College, Beijing, China

**Keywords:** Gastric cancer, Neutrophils, IL-17a, Epithelial mesenchymal transition, Migration and invasion

## Abstract

**Purpose:**

Epithelial to mesenchymal transition (EMT) can contribute to gastric cancer (GC) progression and recurrence following therapy. Tumor-associated neutrophils (TANs) are associated with poor outcomes in a variety of cancers. However, it is not clear whether TANs interact with the EMT process during GC development.

**Methods:**

Immunohistochemistry was performed to examine the distribution and levels of CD66 + neutrophils in samples from 327 patients with GC. CD66b + TANs were isolated either directly from GC cell suspensions or were conditioned from healthy donor peripheral blood polymorphonuclear neutrophils (PMNs) stimulated with tumor tissue culture supernatants (TTCS) and placed into co-culture with MKN45 or MKN74 cells, after which migration, invasion and EMT were measured. Interleukin-17a (IL-17a) was blocked with a polyclonal antibody, and the STAT3 pathway was blocked with the specific inhibitor AG490.

**Results:**

Neutrophils were widely distributed in gastric tissues of patients with GC and were enriched predominantly at the invasion margin. Neutrophil levels at the invasion margin were an independent predictor of poor disease-free survival (DFS) and disease-specific survival (DSS). IL-17a + neutrophils constituted a large portion of IL-17a-producing cells in GC, and IL-17a was produced at the highest levels in co-culture compared with that in TANs not undergoing co-culture. TANs enhanced the migration, invasion and EMT of GC cells through the secretion of IL-17a, which activated the Janus kinase 2/signal transducers and activators of transcription (JAK2/STAT3) pathway in GC cells, while deprivation of IL-17a using a neutralizing antibody or inhibition of the JAK2/STAT3 pathway with AG490 markedly reversed these TAN-induced phenotypes in GC cells induced by TANs.

**Conclusions:**

Neutrophils correlate with tumor stage and predict poor prognosis in GC. TANs produce IL-17a, which promotes EMT of GC cells through JAK2/STAT3 signalling. Blockade of IL-17a signalling with a neutralizing antibody inhibits TAN-stimulated activity in GC cells. Therefore, IL-17a-targeted therapy might be used to treat patients with GC.

**Electronic supplementary material:**

The online version of this article (10.1186/s13046-018-1003-0) contains supplementary material, which is available to authorized users.

## Introduction

Gastric cancer (GC) is the fifth most common malignancy and the third leading cause of cancer-related death worldwide, and the highest mortality rates are seen in East Asia, including China [1, 2]. Although overall survival has improved with the implementation of standard D2 lymphadenectomy and the advancement of chemotherapy and targeted treatments [3–5], the survival rate of patients with GC is less than 30% [2]. Currently, the mechanisms of GC remain unclear. However, recent evidence has indicated that cross-talk between tumor cells and immune or nonimmune stromal cells creates a unique microenvironment that is essential for tumor growth, invasion, and metastasis [6, 7]. The interaction between tumor cells and stromal cells may polarize stromal cells to favour tumor promotion [7].

In addition to tumor cells, a variety of immune stromal cells are the main components of the GC environment. Neutrophils, as the most abundant circulating leucocytes, are also one type of mostly infiltrating immune and inflammatory cells in GC [8]. It has been reported that elevated peripheral blood neutrophils and the neutrophil/lymphocyte ratio predict poor outcomes in many types of cancers, including GC [9–11]. The same has been found in neutrophils that have infiltrated in tumors [9]. In the tumor microenvironment, tumor-associated neutrophils (TANs) have been proposed to promote cancer initiation, progression, and metastasis [12]. In addition to contact-dependent mechanisms, neutrophils may influence tumor progression through the paracrine release of cytokines and chemokines with protumor or antitumor functions, depending on the tumor microenvironment [13]. Moreover, neutrophils can release neutrophil extracellular traps (NETs), which can trap circulating tumor cells in vitro; trapping by NETs is associated with increased formation of micrometastasis in vivo [14, 15]. These novel aspects of neutrophil biology may contribute to GC progression and metastasis. However, direct evidence that supports a role of neutrophils in the immunopathogenesis of human cancers is scarce.

Epithelial-mesenchymal transition (EMT), a well-characterized embryological process, has been identified to play a critical role in tumor progression, invasion and metastasis, and is a way by which cancer cells gain more aggressive properties. In the process of EMT, epithelial cells undergo a phenotypic switch by losing their cell polarity and expression of epithelial markers (E-cadherin, β-catenin), to become mesenchymal cells through the acquisition of mesenchymal markers (N-cadherin, Vimentin, ZEB1) expression; thus, these transformed epithelial cells acquire fibroblast-like properties and exhibit reduced cell-cell adhesion and increased motility [16–18]. The enhanced motility and invasiveness afforded by EMT is critical in the initiation of metastasis for cancer progression, and the acquisition of a mesenchymal phenotype has also been shown to enhance resistance to chemotherapy and lead to a poor prognosis [19, 20]. The expression of these EMT markers can be induced by a number of growth factors/cytokines such as transforming growth factor (TGF)-β, interleukin-6 (IL-6), and CXCL12 [21–23], as well as a variety of transcription factors such as STAT3 and hypoxia-inducible factor-1α (HIF-1α) [22, 24].

Epithelial cell-stromal cell interactions usually regulate EMT, and the factors that induce EMT are often originated from the stromal cells that constitute the tumor microenvironment. Neutrophils are essential components of the tumor stroma and the key players in regulating tumor progression [12, 25]. It has been observed that TANs actively communicate with tumor cells through growth factors or inflammatory cytokines such as TNFα, CCL2, IL-8, and IL-17a, which can promote tumorigenesis and progression [9, 26]. Neutrophils are a source of IL-17a in the setting of inflammation and autoimmune diseases [27, 28]. IL-17a is an immune and inflammatory mediator with multiple biological activities. It is widely found in the inflammatory microenvironment of various tumors, including GC, and is involved in promoting tumor cell migration and invasion, chemotherapy resistance, and immunosuppression, which causes tumor progression and metastasis [29–31]. Clinical studies have suggested that an elevated number of IL-17a-producing cells is an independent marker of adverse survival in cancer [32]. IL-17a exerts its effects by binding to IL-17Ra, a common cytokine receptor, which leads to activation of the Janus kinase (JAK) family of tyrosine kinases and the signal transducers and activators of transcription (STAT) family, particularly STAT3 [33, 34]. Activation of the IL-17a/JAK2/STAT3 pathway plays an active role in the progression of a variety of tumors [29, 33, 35]. Studies have shown that both STAT3 and phosphorylated STAT3 increased in intestinal-type GC compared with normal gastric tissues [36]. Furthermore, phosphorylated STAT3 was positively associated with poorly differentiated adenocarcinoma, lymph node metastasis, and poor prognosis [37]. However, the role of TANs and IL-17a in GC has not been well addressed. Therefore, our aim was to determine how TANs promote the migration and invasiveness of GC cells and to reveal the association between TANs and activation of the IL-17a/JAK2/STAT3 pathway in the progression of GC cells.

In this study, we have shown that neutrophils in GC correlate with prognosis. Neutrophils are a source of IL-17a in clinical sample analysis and experimental studies. We tested the hypothesis that TAN-derived IL-17a enhances migration, invasiveness and EMT of GC cells. We show that EMT is induced by GC neutrophils via secretion of IL-17a and activation of JAK2/STAT3 signalling in GC cells. This effect was blocked by application of an IL-17a neutralizing antibody. Thus, an IL-17a neutralizing antibody could serve as a novel therapeutic strategy in GC.

## Materials and methods

### Patients and clinical specimens

In all, 327 patients who presented between 2007 and 2009 at the Department of Gastroenterological Surgery of Harbin Medical University Cancer Hospital (Harbin, China) were recruited for this study. In this study, all patients were diagnosed with gastric adenocarcinoma, and no patients were treated with neoadjuvant chemotherapy. Patients with infectious diseases, autoimmune disease or multiple primary cancers were excluded from the study. Paraffin-embedded and formalin-fixed tissues were obtained from 327 patients with GC. The medical data and follow-up information of GC patients were identified from our prospective database. Fresh paired intratumoral and nontumoral (at least 5 cm from the tumor site) tissues were obtained from patients with GC who underwent surgical resection, 10 samples were used for immunofluorescence, 10 samples were used for neutrophils isolation, and 10 samples were used to prepare tumor tissue culture supernatants (TTCS) or non-tumor tissue culture supernatants (NTCS). Peripheral blood from 20 healthy donors was also used for neutrophils isolation.

The tumor size was defined according to the longest diameters of the samples. The eighth edition of the American Joint Committee on Cancer Tumor Node Metastasis (AJCC TNM) staging classification for carcinoma of the stomach was used for tumor staging. The Lauren classification was defined as intestinal type, diffuse type, and mixed type. The histological grade was classified as G1, G2, G3 adenocarcinoma, signet ring cell carcinoma, and mucinous adenocarcinoma. Lymphovascular invasion and perineural invasion were diagnosed by H&E-stained slides. Disease-free survival (DFS) was defined as the date of surgery to the date of identification of disease recurrence, which was either radiological or histological. Disease-specific survival (DSS) was calculated from the date of surgery to the date of death from GC; patients who died of causes unrelated to the disease were censored at the last follow-up. This retrospective study was approved by the ethics committee of Harbin Medical University Cancer Hospital, China.

### Immunohistochemistry and immunofluorescence

Immunohistochemical staining was performed using the avidin-biotin-peroxidase complex method. Briefly, paraffin-embedded and formalin-fixed tissues were cut into 4 μm sections and incubated on slides. The sections were deparaffinized in xylene and rehydrated in graded ethanol solutions. Then the slides were incubated in 3% H_2_O_2_ to block the endogenous peroxidase activity. Antigen retrieval was performed by autoclaving the sections for 2 min in citrate buffer (pH 6.0). Primary antibodies against CD66b (1:400 dilution, 555,723, BD) and IL-17a (1:200 dilution, ARG55256, Arigo) were applied to the slides, which were incubated at 4 °C overnight. The Envision-plus detection system was applied to the sections with anti-mouse polymer (1:500 dilution, ab205719, Abcam) or anti-rabbit polymer (1:500 dilution, ab6721, Abcam) at 37 °C for 30 min. Staining was performed with 3,30-diaminobenzidine tetra hydrochloride and counterstaining was performed with Mayer’s haematoxylin. In all assays, we included negative control slides with the primary antibodies omitted. The tissue sections were screened using an inverted research microscope (Nikon, Japan).

For immunofluorescence analysis, frozen sections of human GC tissues were fixed in acetone for 15 min and were permeabilized with 0.1% Triton X-100 for 15 min at room temperature. After blocking with normal nonimmune goat serum for 30 min, tissue sections were incubated with CD66b (1:100 dilution, 555,723, BD) and IL-17a (1:100 dilution, ab9565, Abcam) antibodies. Then, the tissue sections were stained with Alexa Fluor 555-conjugated anti-mouse IgG (A16071, Invitrogen) and Alexa Fluor 488-conjugated anti-rabbit IgG (31,635, Invitrogen) antibodies, Nuclei were stained with 4′,6-diamidino-2-phenylindole (DAPI). Negative control staining was performed by omission of the primary antibody. Immunofluorescence images were observed using a fluorescence microscope equipped with the MetaMorph Imaging System (Universal Imaging Corporation).

### Tumor cell lines

Gastric cell lines (GES-1, MKN45, and MKN74) were purchased from Shanghai Institutes for Biological Sciences, Chinese Academy of Sciences, and were cultured at 37 °C in a humidified atmosphere of 5% CO_2_ in RPMI-1640 medium containing 10% FBS with 100 U/ml penicillin and 100 U/ml streptomycin. For co-culture studies, 12-well Transwell chambers with 0.4 μm porous polycarbonate membranes (Corning, Union City, CA, USA) were used with 1 × 10^6^ MKN45 or MKN74 cells seeded 24 h before co-culture and 2.5–5 × 10^6^ TANs added to the upper or lower chamber. Humanized anti-IL-17a receptor antibody (Abcam, USA, 10 μg/ml) or human IgG antibody (10 μg/ml) were used in the indicated experiments and added to the selected wells. After 24 to 48 h, GC cells and CD66b + TANs cells were collected.

### Neutrophil isolation

#### Neutrophils from peripheral blood

Peripheral blood was collected from healthy donors after written informed consent was obtained. First, 5.0 ml of anti-coagulated whole blood was layered over 5.0 ml of PolymorphPrep (Axis-Shield PoC AS, Oslo, Norway) in a 15 ml centrifuge tube. The samples layered over PolymorphPrep were centrifuged at 500 g for 30 min in a swing-out rotor at room temperature. After centrifugation, two leukocyte bands could be visible. The top band at the sample/medium interface should consist of mononuclear cells, while the lower band should consist of polymorphonuclear neutrophils (PMNs). The cell bands were harvested using a Pasteur pipette. The remnant RBCs were lysed using a hypotonic lysis procedure to obtains a pure PMN population. The morphological examination and cell count were performed to determine the number and purity of the PMNs. Neutrophils were cultured in RPMI-1640 containing 10% FBS with 100 U/ml penicillin and 100 U/ml streptomycin. The purity of the neutrophils was 98% after this procedure. The sorted cells were used unless their viability was determined > 90% and their purity was determined > 95%.

#### Neutrophil from gastric tissues

For neutrophil isolation, fresh gastric tissues were sliced into small pieces and digested in RPMI-1640 supplemented with 0.05% collagenase IV (Sigma-Aldrich, St. Louis, MO), 0.002% DNase I (Roche, Indianapolis, IN), and 20% fetal bovine serum at 37 °C for 60 min. We filtered dissociated cells through a 150 mm mesh and then these cells were centrifuged at 2500 rpm for 20 min at a 1 ml cell suspension and 10 ml of Ficoll-Hypaque (Stemcell Technologies, Vancouver, Canada) in a 15 ml tube. Thereafter, the leukocytes were harvested and CD66b + neutrophils were isolated using the EasySep PE Selection Kit (Stemcell Technologies, Vancouver, Canada) according to the manufacturer’s protocol. We confirmed purification of neutrophils via fluorescence-activated cell sorting analysis with anti-CD66b antibodies, which showed that their viability was greater than 85% and that their purity was greater than 85% for TANs.

#### Preparation of TTCS and NTCS and supernatant-conditioned neutrophils

Tumor tissue culture supernatants (TTCS) or non-tumor tissue culture supernatants (NTCS) were prepared by plating autologous tumor or non-tumor gastric tissues in 1 ml RPMI-1640 containing 10% FBS with 100 U/ml penicillin and 100 U/ml streptomycin for 24 h. The supernatant was then centrifuged and harvested. To generate supernatant-conditioned neutrophils, neutrophils from healthy donors were first harvested and cultured with 30% TTCS or NTCS at a density of 2.5 × 10^6^ cells per ml for 24 h and were then washed with RPMI-1640 medium for three times. Neutrophils cultured with RPMI-1640 medium were used as controls.

#### Enzyme-linked immunosorbent assay (ELISA)

The concentration of IL-17a in the medium was measured by an ELISA (R&D Systems, Minneapolis, MN, USA) according to the manufacturer’s instructions.

#### Cell migration and invasion assay

The cell migration/invasion assay was performed in a 24-well Boyden chamber with an 8-mm pore size polycarbonate membrane (Corning, Union City, CA, USA). An appropriate amount of Matrigel (1:8) was added to the upper chamber of the Transwell plates for the invasion assay, while the plates without Matrigel in the upper chamber were used for the migration assay. The treated cells were incubated in serum-free medium for 24 h and 1.25–2.5 × 10^5^ TANs in 500 μl RPMI-1640 containing 10% FBS were added to the lower chamber before 5 × 10^4^ MKN45 or MKN74 cells in 500 μl in RPMI-1640 containing 10% FBS were added to the upper chamber. The cells were incubated at 37 °C for 24–48 h in a 5% CO2 incubator. Cancer cells remaining on the upper surface of the membrane were removed. The migrated cells on the lower surface of the membrane were rinsed with PBS for 5 min to remove residual neutrophils and were subsequently fixed and stained with crystal violet. Subsequently, at least six randomly selected fields were counted and the average number was presented.

#### Quantitative real-time PCR (qRT-PCR)

Total RNA was extracted using TRIzol reagent (Invitrogen, Carlsbad, CA, USA) according to the manufacturer’s manual. RNA (1 μg) was reverse transcribed into cDNA using a Reverse Transcription System (Promega, Madison, WI, USA). To quantify the IL-17a mRNA level, real-time PCR was performed using a Light Cycler 480 SYBRGreen Kit (Roche Applied Science, Mannheim, Germany) according to the manufacturer’s instructions. GAPDH served as the endogenous reference. Data were analysed by using the comparative Ct method. The specificity of the resulting PCR products was confirmed by examination of the melting curves. The primers used in this assay were:

IL-17a: 5′-CGGTCCAGTTGCCTTCTCCC-3′ (upper)

and 5′-GAGTGGCTGTCTGTGTGGGG-3′ (lower);

GAPDH: 5′-GGACCTGACCTGCCGTCTAG-3′ (upper)

and 5′-GTAGCCCAGGATGCCCTTGA-3′ (lower).

#### Western blot analysis

Total protein was extracted from cells using RIPA buffer in the presence of protease inhibitor (Sigma, USA) and phosphatase inhibitor cocktail (Sigma, USA). The protein concentration was determined with a BCA Protein Assay Kit (Beyotime, Shanghai, China). Proteins were separated by 10% SDS-PAGE and then transferred to polyvinylidene difluoride (PVDF) membranes (Millipore, MA, USA). The membranes were blocked with 5% nonfat milk (BD Biosciences, WA, USA) and 0.1% Tween-20 in Tris-buffered saline and immunoblotted overnight with primary antibodies at 4 °C with gentle shaking. Subsequently, the membranes were stained with HRP-conjugated secondary antibody. Proteins were visualized using ECL Western Blotting Substrate or Super Signal West Femto Chemiluminescent Substrate (Pierce, Rockford, IL, USA) followed by exposure to film. Antibodies used in this study are as follows: Anti-GADPH (1:1000 dilution, ARG10112, Arigo), anti-E-cadherin (1:1000 dilution, ARG66195, Arigo), anti-Vimentin (1:1000 dilution, ARG66199, Arigo), anti-ZEB1 (1:500 dilution, ARG57524, Arigo), anti-p-JAK2 (1:500 dilution, ARG57812, Arigo), anti-JAK2 (1:500 dilution, ARG57629, Arigo), anti-p-STAT3(1:500 dilution, ARG51549, Arigo), and anti-STAT3 (1:500 dilution, ARG53604, Arigo). The secondary goat anti-rabbit or goat anti-mouse (1:5000 dilution, Abcam, USA).

#### Statistical analyses

SPSS 19.0 software (Version 19.0, Chicago, IL, USA) and Graphpad prism 5.0 were used for all statistical analyses. The results were expressed as the mean ± S.D. Analyses of variance and Pearson chi-square tests were used to assess any associations between variables. Clinical outcomes were calculated by Kaplan-Meier survival curves, and the groups were compared using the log-rank test. Stepwise multivariate Cox proportional analysis was also performed. The level of significance permitting multivariate analysis inclusion and the statistical significance for all other tests used was set at *P* < 0.05.

## Results

### The distribution of neutrophils in GC and its relationship with clinicopathological features

In total, 327 patients were enrolled in the study. The clinicopathological features of GC patients are shown in Additional file 1: Table S1. The median DFS and DSS of the GC patients were 25.1 months and 33.9 months, respectively. Moreover, 221 patients (67.7%) experienced postoperative recurrence, and 214 (65.4%) had died of GC by the final follow-up. The median follow-up duration was 45.7 months (range 3.03–112.1 months), and the average age was 57.3 years (range 29–88 years). In addition, 186 of 300 patients received postoperative adjuvant chemotherapy, and the average number of harvested lymph nodes after surgical resection was 37.5 (range 10–69).

**Fig. 1:**
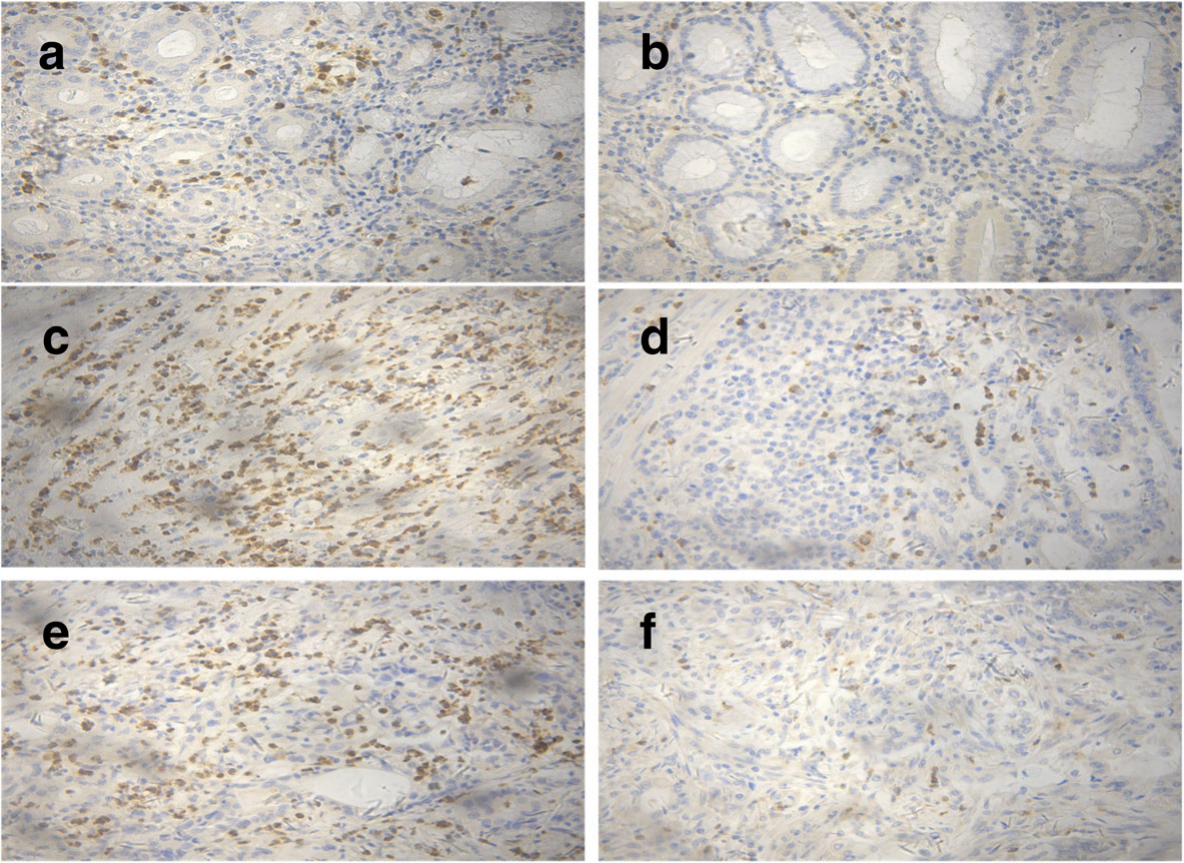
Representative picture of immunohistochemical staining of CD66b + neutrophils at the nontumoral, invasive margin and tumor center tissues of GC sampers. **a** High CD66b + neutrophils density at the nontumoral gastric tissues. **b** Low CD66b + neutrophils density at the nontumoral gastric tissues. **c** High CD66b + neutrophils density at the invasive margin of GC tissues. **d** Low CD66b + neutrophils density at the invasive margin of GC tissues. **e** High CD66b + neutrophils density at the tumor center of GC tissues. **f** Low CD66b + neutrophils density at the tumor center of GC tissues. Magnifications: × 200

Neutrophils were widely distributed in the gastric tissues of patients with GC and were obviously increased in number in GC tissues, especially, at the invasion of the edge. The number of neutrophils at the invasion margin was significantly higher than that at the nontumoral tissues (101.70 ± 3.3 vs 8.46 ± 0.49, *P* < 0.001) and that at the tumor center (101.70 ± 3.3 vs 59.96 ± 2.22, *P* < 0.001) (Fig. 1). No correlation was observed between the number of neutrophils at the nontumoral tissues and the clinicopathological features of GC (all *P* > 0.05) (Additional file 1: Table S1). The number of neutrophils at the invasion margin was significantly associated with TNM stage (*P* = 0.016), lymphovascular invasion (*P* = 0.008), and perineural invasion (*P* < 0.001). However, the number of neutrophils was not associated with age, gender, tumor location, tumor size, Lauren type, or histological grade (Additional file 1: Table S1). The number of neutrophils at the tumor center was correlated with lymphovascular invasion (*P* < 0.001), and perineural invasion (*P* < 0.025), but was not associated with age, gender, tumor location, tumor size, TNM stage, Lauren type, or histological grade (Additional file 1: Table S1).

### The relationship between neutrophils in GC and prognosis

**Fig. 2:**
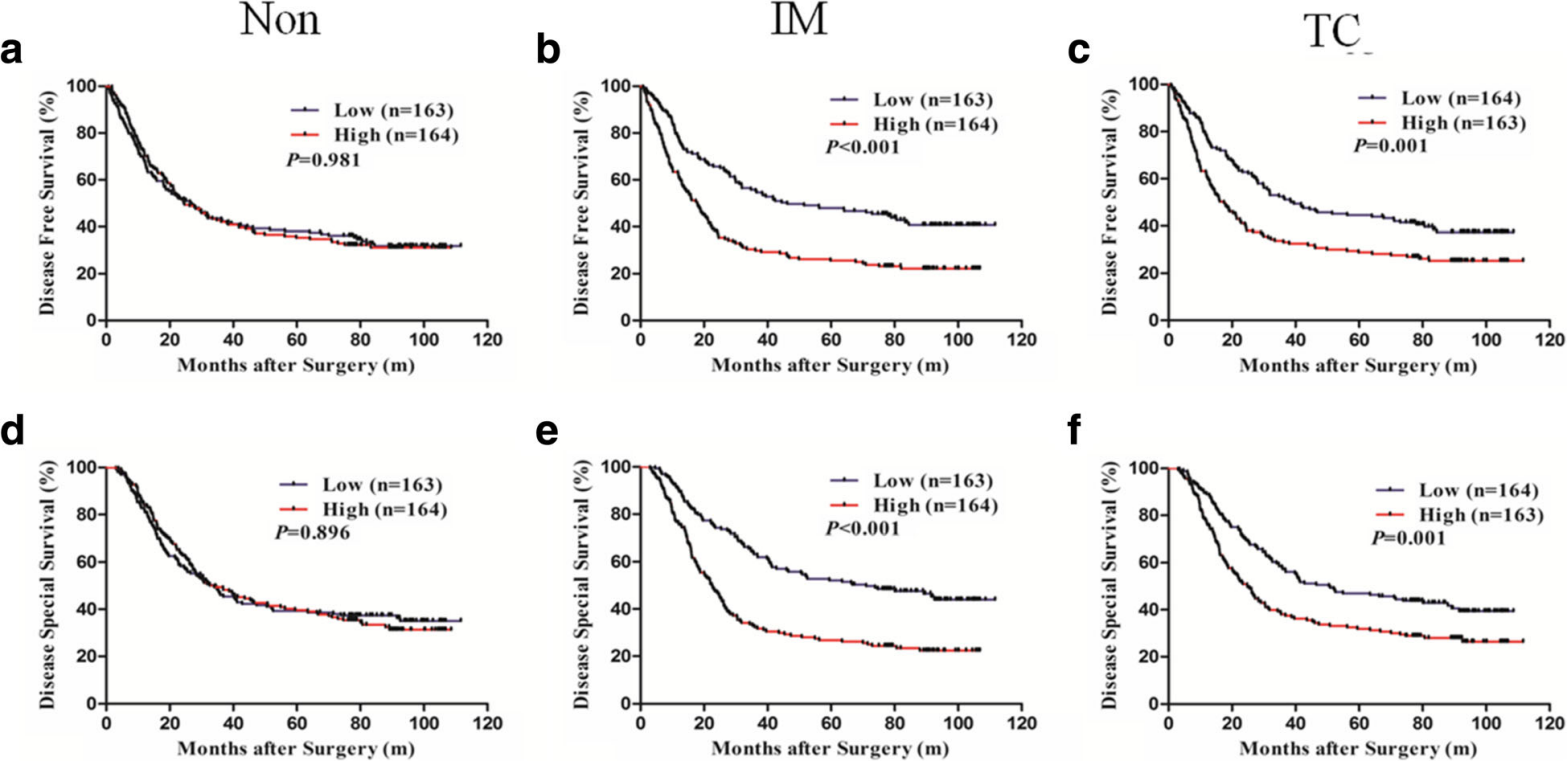
Kaplan-Meier curves of DFS and DSS based on the number of neutrophils at the nontumoral, invasive margin and tumor center tissues of patients with GC. **a**, **d** Higher number of neutrophils at the nontumoral tissues were not correlated with prognosis (*P* = 0.981 and *P* = 0.896). **b**, **e** Higher number of neutrophil at the invasive margin of GC tissues were closely correlated with poor DFS and DSS (*P* = 0.001 and *P* = 0.001). **c**, **f** Higher number of neutrophil at the tumor center of GC tissues were closely correlated with poor DFS and DSS (*P* < 0.001 and *P* < 0.001)

The univariate analysis indicated that the number of neutrophils at the nontumoral tissues was not associated with DFS or DSS (*P* = 0.981 and *P* = 0.896), but the number of neutrophils at the invasion margin and at the tumor center all demonstrated a statistically significant association with DFS and DSS (*P* = 0.001 and *P* < 0.001, *P* = 0.001 and *P* < 0.001, respectively) (Additional file 2: Table S2 and Additional file 3: Table S3) (Fig. 2). In addition, ASA (*P* = 0.002), tumor site (*P* = 0.022), tumor size (*P* < 0.001), TNM stage (*P* < 0.001), lymphovascular invasion (*P* = 0.002), perineural invasion (*P* < 0.001), and postoperative chemotherapy (*P* = 0.032) all demonstrated a statistically significant association with DFS, whereas age, gender, Lauren type, and histological grade had no prognostic significance for DFS (Additional file 2: Table S2). ASA (*P* = 0.001), tumor site (*P* = 0.029), tumor size (*P* < 0.001), TNM stage (*P* < 0.001), lymphovascular invasion (*P* = 0.001), perineural invasion (*P* < 0.001), and postoperative chemotherapy (*P* = 0.010) were all significantly associated with patients with DSS, whereas age, gender, Lauren type, and histological grade had no prognostic significance for DSS (Additional file 3: Table S3).

The multivariate analysis showed that TNM stage (*P* < 0.001), perineural invasion (*P* = 0.015), postoperative chemotherapy (*P* = 0.006), and CD66bIM (*P* = 0.001) were independent risk factors for DFS in GC (Additional file 2: Table S2). In addition, TNM stage (*P* < 0.001), perineural invasion (*P* = 0.013), postoperative chemotherapy (*P* = 0.001), and CD66bIM (*P* < 0.001) were independent risk factors for DSS in GC (Additional file 3: Table S3). The results of the comprehensive analysis showed that higher TNM stage, perineural invasion, lack of postoperative chemotherapy, and CD66bIM were independent risk factors for the prognosis of GC.

### IL-17a protein is primarily expressed by neutrophils in GC

Neutrophils play an important role in promoting tumor progression in GC. But the role of IL-17a in tumors is still unclear. Although the role of IL-17a in lymphocytes has been widely studied, recent studies have found that IL-17a is also expressed in other immune inflammatory cells, including neutrophils, but the association between neutrophils and IL-17a + cells is unknown. Therefore, we assessed the association between neutrophils and IL-17a + cells in GC, with a specific focus on tissue micro-location of the cells.

**Fig. 3:**
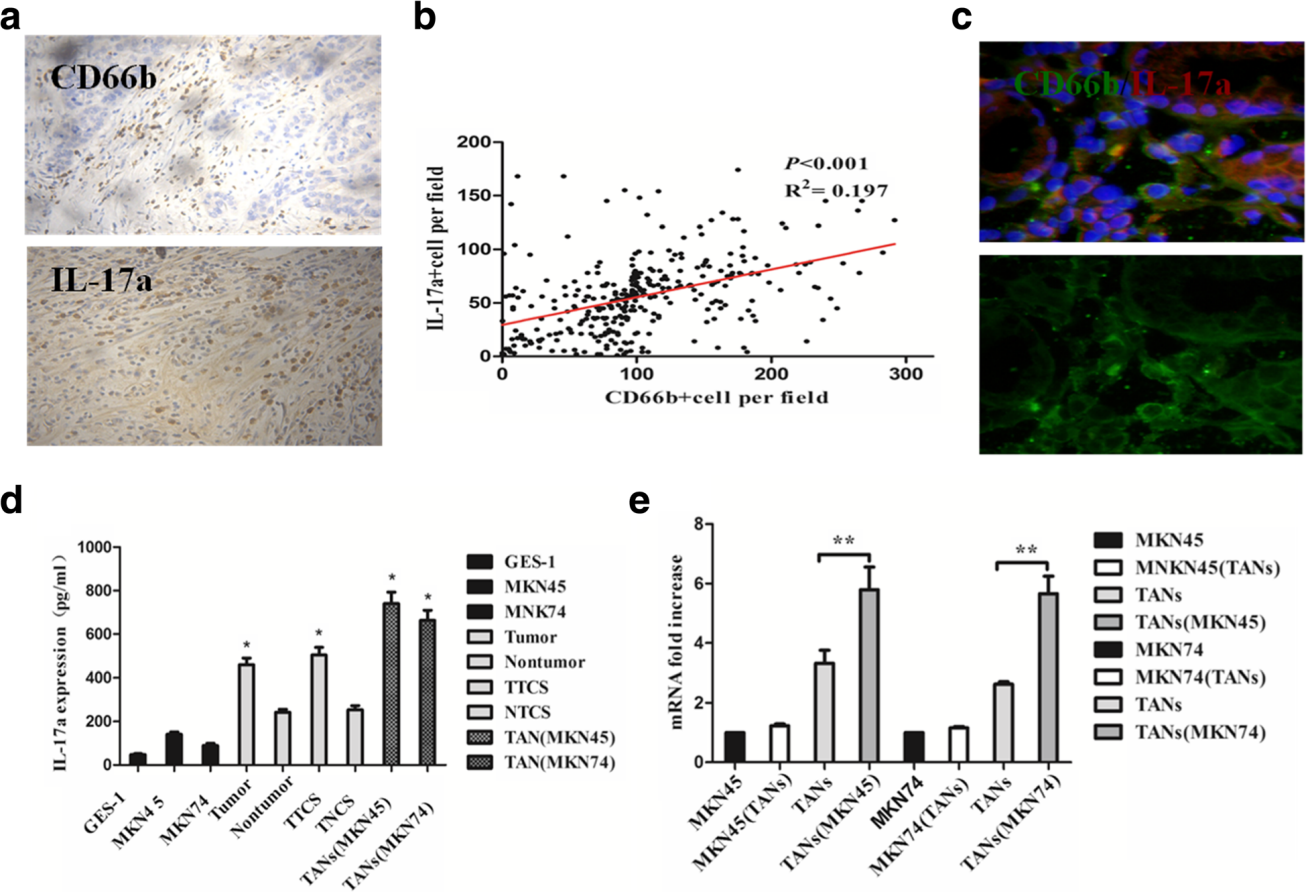
IL-17a protein is primarily expressed by neutriphils in GC. **a**, **b** Association of CD66b + neutriphils and IL-17a + cells at the invasive margin of GC tissues by immunohistochemical staining. Magnifications: × 200. **c** Analysis of IL-17a and CD66b distribution in GC tissues by immunofluorescence microscope. One of 10 representative micrographs is shown. Magnifications: × 400. **d** The production of IL-17a in the cell supernatant in the gastric cell lines (GES-1, MKN45 and MK74), the production of IL-17a in the cell supernatant in the neutrophils isolated from GC tissues and nontumoral tissues, neutrophils activated by TTCS and NTCS, the production of IL-17a in the co-culture system were quantified 24 h after change the culture medium by ELISA. * *P* < 0.05. **e** the expression level of IL-17a mRNA in the GC cells (MKN45 and MK74), GC cells co-cultured neutrophils, neutrophils, and neutrophils co-cultured GC cells were quantified 24 h after change the culture medium by qRT-PCR. ** *P* < 0.001

Immunohistochemical staining showed that IL-17a + cells were mainly distributed in the peritumoral stroma (Fig. 3a) and that IL-17a was negatively related to DFS and DSS (*P* < 0.001 and *P* < 0.001) (Additional file 4: Figure S1). Both CD66b + neutrophils and IL-17a + cells were observed in the same area (R^2^ = 0.155, *P* < 0.001) (Fig. 3b). Immunofluorescence further found that (61.6 ± 7.5) % of IL-17a protein was expressed by CD66b + neutrophils in the tumor stroma which was significantly higher than that in the nontumoral tissues (15.4 ± 5.8)% (*P* < 0.001) (Fig. 3c). Therefore, neutrophils are the primary cells that produce IL-17a.

The production of IL-17a in the cell supernatant of the gastric cell lines GES-1, MKN45, and MKN74 was 47.67 + 4.26 pg/ml, 141.7 ± 10.41 pg/ml, and 89.33 + 10.35 pg/ml. The production of IL-17a in the cell supernatant of neutrophils isolated from GC tissues and nontumoral tissues and neutrophils activated by TTCS and NTCS was 458.3 ± 31.14 pg/ml, 242.0 ± 14.05 pg/ml, 503.3 ± 35.63 pg/ml, and 254.07 ± 19.29 pg/ml, respectively. The production of IL-17a in the co-culture system was 740.3 ± 52.92 pg/ml and 663.3 ± 46.31 pg/ml, respectively (Fig. 3d).

The ELISA results showed that gastric cell lines (GES-1, MKN45 and MKN74) produced a low level of IL-17a and that neutrophils isolated from GC tissues produced a higher level of IL-17a than neutrophils isolated from nontumoral tissues (*P* < 0.05). Moreover, neutrophils activated by TTCS produced a higher level of IL-17a than neutrophils activated by NTCS (*P* < 0.05), and the co-culture system produced higher levels of IL-17a (*P* < 0.05) (Fig. 3d).

In addition, qRT-PCR results also showed that the expression level of IL-17a mRNA in GC cells (MKN45 and MKN74) increased slightly after co-culture with neutrophils, while the expression level of IL-17a mRNA in neutrophils increased significantly after co-culture with GC cells (MKN45 and MKN74) (*P* < 0.001 and *P* < 0.001) (Fig. 3e). Neutrophils in the tumor microenvironment may be the main source of IL-17a.

### Neutrophils promote migration and invasiveness and EMT of GC cells through IL-17a

**Fig. 4:**
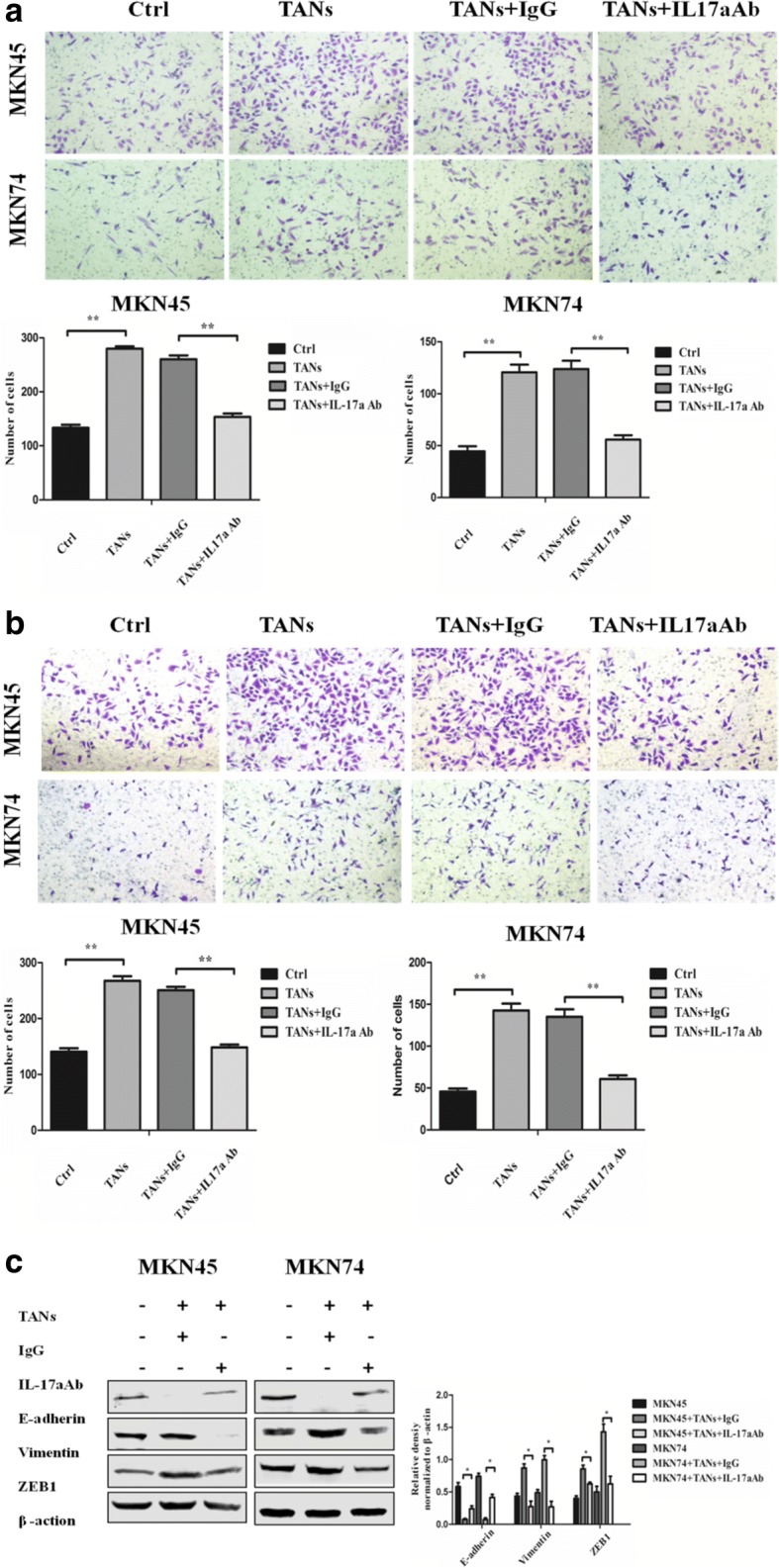
Neutrophils enhance the migration, invasiveness and EMT of GC cells through IL-17a. (**a**, **b**) The effect of neutrophils on the migration and invasion ability of GC cells (MKN45 and MKN74) was determined 24 h when IL-17a neutralizing antibody or IgG isotype control antibody was added to Transwell co-culture chamber. Magnifications: × 100. *, *P* < 0.05; **, *P* < 0.001. **c** Protein expression of E-cadherin, Vimentin, and ZEB1 in GC cells (MKN45 and MKN74) co-cultured with neutrophils were analyzed by western blot when IL-17a neutralizing antibody or IgG isotype control antibody was added to the Transwell co-culture system. Densitometric analysis of E-cadherin, Vimentin, and ZEB1 expression were shown. **, *P* < 0.001

To study the effect of neutrophils on GC cells, a Transwell migration/invasion assay was used to explore the effect of neutrophils on the migration and invasion ability of GC cells. The results showed that neutrophils promote GC cell (MKN45 and MKN74) migration (*P* < 0.001 and *P* < 0.001) and invasion (*P* < 0.001 and *P* < 0.001) (Fig. 4a and b), when an IL-17a neutralizing antibody was added to the Transwell co-culture chamber, GC cell (MKN45 and MKN74) migration (*P* < 0.001 and *P* < 0.001) and invasion were decreased (*P* < 0.001 and *P* < 0.001) (Fig. 4a and b). Therefore, neutrophil promotes the migration and invasiveness of GC cells through IL-17a.

EMT, a well-characterized embryological process, has been identified to play a critical role in tumor metastasis. This process is characterized by loss of epithelial markers (e.g. E-cadherin) and the acquisition of mesenchymal markers (e.g. Vimentin, ZEB1). To examine the role of TANs in mediating EMT in GC cells, we cultured GC cells (MKN45 and MKN74) with TANs in a previously described co-culture system.

Western blot results showed that neutrophils promote the expression of EMT related markers in GC cells (MKN45 and MKN74). E-cadherin expression was significantly decreased (*P* < 0.05 and *P* < 0.05 in MKN45 and MKN74 cells, respectively), and Vimentin (*P* < 0.05 and *P* < 0.05 in MKN45 and MKN74 cells, respectively) and ZEB1 (*P* < 0.05 and *P* < 0.05 in MKN45 and MKN74 cells, respectively) expression was significantly upregulated (Fig. 4c), when an IL-17a neutralizing antibody was added to the Transwell co-culture system, The expression of E-cadherin protein in the epithelium of GC cells was upregulated (*P* < 0.05 and *P* < 0.05 in MKN45 and MKN74 cells, respectively), while the expression of Vimentin protein in the interstitial space decreased significantly (*P* < 0.05 and *P* < 0.05 in MKN45 and MKN74 cells, respectively). The expression of the transcription factor ZEB1 was also significantly downregulated (*P* < 0.05 and *P* < 0.05 in MKN45 and MKN74 cells, respectively) (Fig. 4c). Therefore, neutrophils promote EMT in GC cells, and further promotes the migration and invasiveness of GC cells, which may be mediated by IL-17a.

### IL-17a activates the STAT3 signalling pathway and promotes EMT and migration and invasiveness of GC cells

**Fig. 5:**
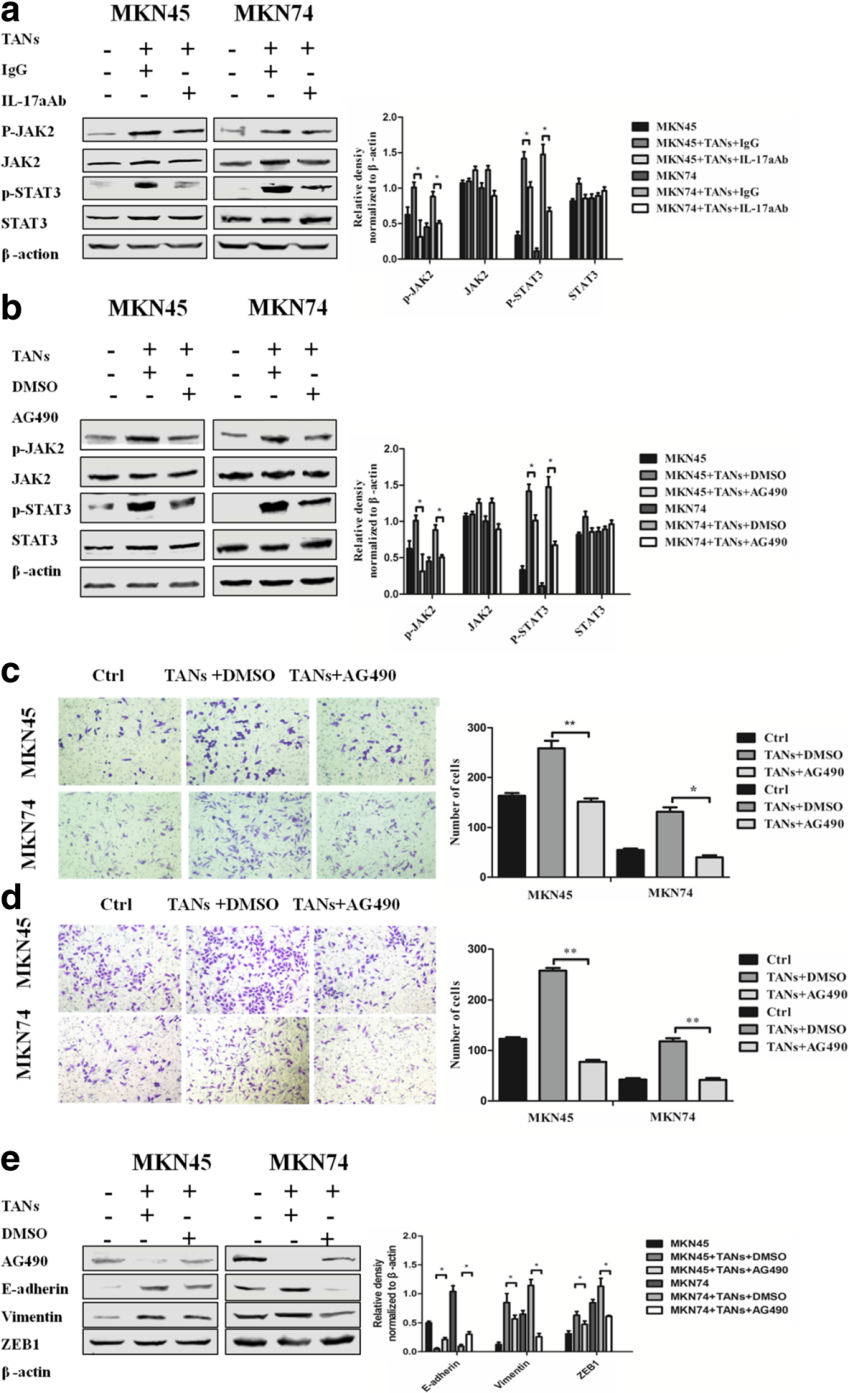
IL-17a activates the STAT3 signalling pathway and promotes EMT, migration and invasiveness of GC cells. **a**, **b** Protein expression of p-JAK2, JAK2, p-STAT3, and STAT3 in GC cells (MKN45 and MKN74) co-cultured with neutrophils were analyzed by western blot when IL-17a neutralizing antibody or AG490 was added to the Transwell co-culture system. Densitometric analysis of p-JAK2, JAK2, p-STAT3, and STAT3 expression were shown. *, *P* < 0.05. **c**, **d** The effect of neutrophils on the migration and invasion ability of GC cells (MKN45 and MKN74) were determined when AG490 was added to the Transwell co-cultured chamber. Magnifications: × 100. *, *P* < 0.05; **, *P* < 0.01. **e** Protein expression of E-cadherin, Vimentin, and ZEB1 in GC cells (MKN45 and MKN74) co-cultured with neutrophils were analyzed when AG490 was added to Transwell co-culture system. Densitometric analysis of E-cadherin, Vimentin, and ZEB1 expression were shown. *, *P* < 0.05

The canonical IL-17a signal transduction pathway is initiated by its binding to IL-17Ra and phosphorylation of STAT3 through JAK2 activation. To determine the role of the IL-17a/JAK2/STAT3 pathway in mediating TANs-induced migration, invasion and EMT of GC cells (MKN45 and MKN74), we first explored the activation of the IL-17a/JAK2/STAT3 pathway in GC cells after co-culture with TANs. Western blot results showed that neutrophils promoted the phosphorylation of JAK2 (*P* < 0.05 and *P* < 0.05 in MKN45 and MKN74 cells, respectively) and STAT3 (*P* < 0.05 and *P* < 0.05 in MKN45 and MKN74 cells, respectively) but had no significant effect on the expression of JAK2 and STAT3 in GC cells (MKN45 and MKN74) (Fig. 5a and b). In contrast, the addition of an IL-17a neutralizing antibody or the JAK2 protein tyrosine kinase inhibitor AG490 to the co-culture system significantly reversed the TANs-mediated phosphorylation of JAK2 (*P* < 0.05 and *P* < 0.05 in MKN45 and MKN74 cells, respectively) and STAT3 (*P* < 0.05 and *P* < 0.05 in MKN45 and MKN74 cells, respectively) and had no effect on the expression of JAK2 and STAT3 in GC cells (MKN45 and MKN74) (Fig. 5a and b). A Transwell migration/invasion assay showed that AG490 decreased GC cell migration (*P* < 0.001 and *P* < 0.05 in MKN45 and MKN74 cells, respectively) and invasiveness (*P* < 0.001 and *P* < 0.05 in MKN45 and MKN74 cells, respectively) (Fig. 5c and d). Western blot analysis showed that AG490 inhibited the expression of EMT-related markers in GC cell, as demonstrated by increased expression of E-cadherin (*P* < 0.05 and *P* < 0.05 in MKN45 and MKN74 cells, respectively) and significantly decreased expression of Vimentin (*P* < 0.05 and *P* < 0.05 in MKN45 and MKN74 cells, respectively) and ZEB1 (*P* < 0.05 and *P* < 0.05 in MKN45 and MKN74 cells, respectively) (Fig. 5e). The IL-17a/JAK2/STAT3 pathway plays an important role in TANs-induced migration, invasiveness and EMT of GC cells.

## Discussion

TANs, which are activated neutrophils in the tumor stroma, are an essential component of the tumor microenvironment and play a role in tumor progression, therefore, they should be appraised carefully [12]. In this study, we showed that neutrophils were highly enriched within GC and that neutrophils in the invasion margin of GC tissues were negatively correlated with patient survival. In addition, we demonstrate that high levels of IL-17a were present in GC tissues and that neutrophils produced IL-17a. In an in vitro experiment, we observed that IL-17a secreted by TANs plays an important role in the progression of GC. TANs-derived-IL-17a promoted the migration, invasiveness and EMT of GC cells via the activation of the JAK2/STAT3 pathway. Inhibition of this pathway with IL-17a an neutralizing antibody or the JAK2-specific inhibitor AG490 reversed these TAN-induced phenotypes in GC cells. Therefore, the activation of the JAK2/STAT3 pathway by IL-17a may play a central role in the interplay between TANs and GC cells.

Importantly, our findings also revealed the clinical relevance of neutrophils in GC. Specifically, we found that an increased frequency of intratumoral neutrophils predicted a poor prognosis. Given that the clinical outcomes of patients with GC remains poor and that few prognostic factors currently exist for this disease following surgery [38], intratumoral neutrophil cell frequency might prove to be a useful clinical marker in the future. Moreover, neutrophils may influence tumor progression through the paracrine release of cytokines and chemokines with protumor or antitumor functions, depending on the tumor microenvironment [13]. Previous studies have shown that neutrophils can produce IL-17a in inflammatory and autoimmune diseases [27, 28]. In addition, neutrophils can promote angiogenesis through IL-17a in GC. In the present study, both clinical samples analysis and an experimental study suggested that IL-17a is predominantly expressed by neutrophils.

Epithelial to mesenchymal transition (EMT) is a process by which epithelial tumor cells lose their epithelial features and gain a mesenchymal phenotype [39]. EMT is considered as the key step by which tumor cells gain the higher ability of invasive and metastatic abilities. Tumor cells take advantage of EMT as an intermediary phenotype to achieve self-renewal and to adapt to their microenvironments [40, 41]. Experimentally, EMT can be induced not only by loss of cellular contact (for example, due to degradation of basement membranes or other modifications of the microenvironment), but also by numerous cytokines, especially by TGF-β [42]. TANs can induce EMT in intratumoral cancer cells, but the molecular mechanisms are poorly understood. Studies have shown that loss of surface-associated E-cadherin, at least in part due to cleavage by neutrophil-derived elastase and the subsequent weakening of the cell-to-cell contacts by loss of cell polarity, is a crucial step in the EMT transition process [42]. In addition, a plethora of factors that are abundant in infiltrated cells that infiltrate the tumor microenvironment (TANs in particular) such as IL-6, IL-8, IL-1β, and TNFα [43] are also able to induce EMT in GC. However, very little is currently known about the mechanisms underlying the polarization of IL-17a + neutrophils and their role in GC progression. In the present study, we have shown that IL-17a secreted by TANs induced EMT of GC cells; this process is characterized by loss of the epithelial markers E-cadherin and the acquisition of the mesenchymal markers Vimentin and ZEB1. Then these EMT changes then contribute to the enhanced capability for active locomotion of GC cells, which is demonstrated by increased migratory ability triggered by TANs.

IL-17 is positively correlated with the degree of activation of STAT3 signalling pathway activation [44]. The activation of STAT3 with the phosphorylation of Tyr705 is facilitated by the JAK signalling pathway [33, 45]. Accumulating evidences shows that activation of the IL-17a/JAK2/STAT3 signalling pathway by growth factors or cytokines plays an active role in tumor growth and progression. However, the role of TANs and IL-17a in GC has not been well addressed. Our present study has shown that TANs induced the phosphorylation of JAK2 and STAT3 in GC cells via the secretion of IL-17a. Our study also showed that inhibiting JAK2/STAT3 pathway activation with AG490 significantly impaired TAN-induced migration and invasion, as well as EMT of GC cells induced by TANs in vitro. TANs are known to secrete multiple growth factors and chemokines such as TNFα, CCL2, IL-8, and IL-17a into the tumor microenvironment, where they promote the growth and invasion of the underlying tumor by triggering multiple pathways [9, 26]. In the present study, we found that an IL-17a neutralizating antibody partly suppressed the JAK2 or STAT3 phosphorylation, which suggested that IL-17a partially contributed to the tumor-promoting effects of TANs on GC cells. Although we can not preclude the likely involvement of other growth factors and/or cytokines, the studies of neutralizing IL-17a or the inhibition of JAK2/STAT3 pathway activation with AG490 reveal that IL-17a is an important mediator of the tumor-promoting effects of TANs, which promote EMT via the activation of the JAK2/STAT3 signalling pathway in GC.

## Conclusions

In summary, we find that neutrophils are highly enriched within GC, and are negatively correlated with patient survival and are associated with disease progression. In vitro data established a link between TANs and EMT of GC cells through IL-17a/JAK2/STAT3 signalling. Therefore therapies that target IL-17a or STAT3 signalling may provide future treatment efficacy in GC and are thus important for clinical study.

## Additional files

Additional file 1: **Table S1** Association of CD66b+cells with clinicopathological feathers in Non, IM and TC of gastric cancer (DOCX 19 kb)

Additional file 2: **Table S2** Univariate and Multivariate analyses of factors Associated with Disease-free Survival (DFS) with gastric adenocarcinoma (DOCX 16 kb)

Additional file 3: **Table S3** Univariate and Multivariate analyses of factors Associated with Disease Special Survival (DSS) with gastric adenocarcinoma (DOCX 16 kb)

Additional file 4: **Figure S1**. Kaplan-Meier curves of DFS and DSS based on the number of IL-17a+cells in GC. (a, b) Higher number of 17a+cells in GC tissues were closely correlated with poor DFS and DSS (*P* < 0.001 and *P* < 0.001). (DOCX 144 kb)

## Abbreviations

DAPI: 4′,6-diamidino-2-phenylindole
DFS: Disease-free survival
DSS: Disease-specific survival
ELISA: Enzyme-linked immunosorbent assay
EMT: Epithelial mesenchymal transition
GC: Gastric cancer
HIF-1α: Hypoxia-inducible factor-1α
IL-17a: Interleukin-17a
IL-6: Interleukin-6
JAK2/STAT3: Janus kinase 2/signal transducers and activators of transcription
JAKs: Janus kinases
NETs: Neutrophil extracellar traps
NTCS: Non-tumor tissue culture supernatants
PMN: Polymorphonuclear
QRT-PCR: Quantitative real-time PCR
STAT: Signal transducers and activators of transcription
TANs: Tumor-associated neutrophils
TGF-β: Transforming growth factor
TTCS: Preparation tumor tissue culture supernatants
