# Correction to: Tumor-associated neutrophils induce EMT by IL-17a to promote migration and invasion in gastric cancer cells

**DOI:** 10.1186/s13046-019-1168-1

**Published:** 2019-04-26

**Authors:** Sen Li, Xiliang Cong, Hongyu Gao, Xiuwen Lan, Zhiguo Li, Wenpeng Wang, Shubin Song, Yimin Wang, Chunfeng Li, Hongfeng Zhang, Yuzhou Zhao, Yingwei Xue

**Affiliations:** 1 Department of Gastroenterological Surgery, Harbin Medical University Cancer Hospital, 150 Ha Ping Road, Harbin, 150081 China; 2 Department of General Surgery, The Affiliated Cancer Hospital of Zhengzhou University, 127 Dong Ming Road, Zhengzhou, 450008 China; 3 Department of Gynecologic Oncology, Cancer Hospital Chinese Academy of Medical Sciences & Peking Union Medical College, Beijing, China

**Correction to: J Exp Clin Cancer Res (2019) 38: 6**

**https://doi.org/10.1186/s13046-018-1003-0**

In the publication of this article [1], there is an error in the order of the authors and the affiliations. The revised order of the authors and affiliations has now been included in this correction.

The corrected correspondence author order and author affiliation information is as given hereafter:

Sen Li^1,2^, Xiliang Cong^1^, Hongyu Gao^1^, Xiuwen Lan^1^, Zhiguo Li^1^, Wenpeng Wang^3^, Shubin Song^1^, Yimin Wang^1^, Chunfeng Li^1^, Hongfeng Zhang^1^, Yuzhou Zhao^2*^ and Yingwei Xue^1*^

1 Department of Gastroenterological Surgery, Harbin Medical University Cancer Hospital, 150 Ha Ping Road, Harbin 150081, China.

2 Department of General Surgery, The Affiliated Cancer Hospital of Zhengzhou University, 127 Dong Ming Road, Zhengzhou 450008, China.

3 Department of Gynecologic Oncology, Cancer Hospital Chinese Academy of Medical Sciences & Peking Union Medical College, Beijing, China.
