# Supplementary material for: Tumor-associated neutrophils induce EMT by IL-17a to promote migration and invasion in gastric cancer cells
Source: J Exp Clin Cancer Res. 2019 Jan 7;38:6. doi: 10.1186/s13046-018-1003-0 (PMC6323742; doi:10.1186/s13046-018-1003-0)
Supplement: Supplementary file 1 — Table S1 Association of CD66b+cells with clinicopathological feathers in Non, IM and TC of gastric cancer (DOCX 19 kb) [file 13046_2018_1003_MOESM1_ESM.docx]

| feathers | CD66b Non | | |  | CD66b IM | | |  | CD66bTC | | |
| --- | --- | --- | --- | --- | --- | --- | --- | --- | --- | --- | --- |
|  | Low N(%) | High N(%) | *P* value |  | Low N(%) | High N(%) | *P* value |  | Low N(%) | High  (%) | *P* value |
| Age |  |  | 0.144 |  |  |  | 0.516 |  |  |  | 0.358 |
| <70 | 133 | 125 |  |  | 127 | 131 |  |  | 132 | 126 |  |
| ≥70 | 30 | 39 |  |  | 37 | 32 |  |  | 31 | 38 |  |
| Gender |  |  | 0.437 |  |  |  | 0.129 |  |  |  | 0.831 |
| Male | 115 | 122 |  |  | 125 | 112 |  |  | 119 | 118 |  |
| Female | 48 | 42 |  |  | 39 | 51 |  |  | 44 | 46 |  |
| ASA status |  |  | 0.900 |  |  |  | 0.862 |  |  |  | 0.185 |
| <3 | 137 | 137 |  |  | 138 | 136 |  |  | 141 | 133 |  |
| ≥3 | 26 | 27 |  |  | 26 | 27 |  |  | 22 | 31 |  |
| Tumor site |  |  | 0.473 |  |  |  | 0.454 |  |  |  | 0.757 |
| Upper | 18 | 24 |  |  | 23 | 19 |  |  | 19 | 23 |  |
| Middle | 58 | 50 |  |  | 49 | 59 |  |  | 53 | 55 |  |
| lower | 87 | 90 |  |  | 92 | 85 |  |  | 91 | 86 |  |
| Tumor size |  |  | 0.864 |  |  |  | 0.993 |  |  |  | 0.348 |
| <5 | 73 | 75 |  |  | 75 | 76 |  |  | 78 | 70 |  |
| ≥5 | 90 | 89 |  |  | 89 | 90 |  |  | 85 | 94 |  |
| Pathological TNM stage |  |  | 0.130 |  |  |  | 0.257 |  |  |  | 0.016 |
| I | 27 | 23 |  |  | 27 | 23 |  |  | 28 | 22 |  |
| II | 30 | 48 |  |  | 44 | 34 |  |  | 48 | 30 |  |
| III | 82 | 75 |  |  | 77 | 80 |  |  | 73 | 84 |  |
| IV | 24 | 18 |  |  | 16 | 26 |  |  | 14 | 28 |  |
| Lauren classification |  |  | 0.202 |  |  |  | 0.654 |  |  |  | 0.353 |
| Intestinal | 83 | 98 |  |  | 87 | 94 |  |  | 87 | 94 |  |
| Diffuse | 30 | 29 |  |  | 30 | 29 |  |  | 27 | 32 |  |
| Mixed | 50 | 37 |  |  | 47 | 40 |  |  | 49 | 38 |  |
| Histological grade |  |  | 0.271 |  |  |  | 0.236 |  |  |  | 0.520 |
| G1/ G2 | 56 | 66 |  |  | 56 | 66 |  |  | 58 | 64 |  |
| G3/Signet ring cell/ Mucinous | 107 | 98 |  |  | 108 | 97 |  |  | 105 | 100 |  |
| Lymphovascular invasion |  |  | 0.972 |  |  |  | <0.001 |  |  |  | 0.008 |
| No | 117 | 118 |  |  | 92 | 55 |  |  | 128 | 107 |  |
| Yes | 46 | 46 |  |  | 72 | 108 |  |  | 35 | 57 |  |
| Perineural invasion |  |  | 0.293 |  |  |  | 0.025 |  |  |  | <0.001 |
| No | 78 | 69 |  |  | 127 | 108 |  |  | 94 | 53 |  |
| Yes | 85 | 95 |  |  | 37 | 55 |  |  | 69 | 111 |  |
| 5-Fu^a^ |  |  | 0.611 |  |  |  | 0.163 |  |  |  | 0.408 |
| Yes | 89 | 97 |  |  | 97 | 89 |  |  | 94 | 92 |  |
| No | 58 | 56 |  |  | 50 | 64 |  |  | 52 | 62 |  |

**Table-1. Association of CD66b+cells with clinicopathological feathers in Non, IM and TC of gastric cancer**

Note: ^a^ Fluoropyrimidine-based adjuvant chemotherapy, mostly including capecitabine plus platinum, capecitabine alone, or S1 (combined tegafur, gimeracil, and oteracil), in patients at advanced stage or early stage tumors with lymph node metastasis in this retrospective study.

Abbreviations: Non: Nontumoral tissues; IM: invasive margin; TC: tumor center; ASA: American Society of Anesthesiology.
