# Supplementary material for: Tumor-associated neutrophils induce EMT by IL-17a to promote migration and invasion in gastric cancer cells
Source: J Exp Clin Cancer Res. 2019 Jan 7;38:6. doi: 10.1186/s13046-018-1003-0 (PMC6323742; doi:10.1186/s13046-018-1003-0)
Supplement: Supplementary file 3 — Table S3 Univariate and Multivariate analyses of factors Associated with Disease Special Survival (DSS) with gastric adenocarcinoma (DOCX 16 kb) [file 13046_2018_1003_MOESM3_ESM.docx]

**Table-3. Univariate and Multivariate analyses of factors Associated with Disease Special Survival (DSS) with gastric adenocarcinoma**

|  | Univariate |  |  | Multivariate |  |
| --- | --- | --- | --- | --- | --- |
| Factors | HR (95% CI) | *P* value |  | HR (95% CI) | *P* value |
| Age (≥70 vs. <70) | 1.324(0.968-1.811) | 0.078 |  |  |  |
| Gender (Femal vs. Male) | 1.077(0.798-1.454) | 0.626 |  |  |  |
| ASA status (≥3 vs. <3) | 1.748(1.253-2.439) | 0.001 |  | 1.717(1.206-2.444) | 0.589 |
| Tumor site |  | 0.029 |  |  | 0.599 |
| Upper | 1 |  |  | 1 | 0.827 |
| Middle | 1.110(0.725-1.699) |  |  | 1.256(0.807-1.955) |  |
| Lower | 0.756(0.502-1.414) |  |  | 1.185(0.775-1.811) |  |
| Tumor size (≥5 vs. <5) | 2.147(1.622-2.842) | <0.001 |  | 1.035(0.761-1.407) |  |
| TNM stage |  | <0.001 |  |  | <0.001 |
| I | 1 |  |  | 1 |  |
| II | 2.905(1.447-5.832) |  |  | 2.674(1.044-6.853) |  |
| III | 7.714(4.041-14.729) |  |  | 6.373(2.544-15.964) |  |
| IV | 18.890(9.376-38.055) |  |  | 18.020(6.815-47.645) |  |
| Lauren classification |  | 0.301 |  |  |  |
| Intestinal | 1 |  |  |  |  |
| Diffuse | 1.292(0.909-1.837) |  |  |  |  |
| Mixed | 0.937(0.704-1.345) |  |  |  |  |
| Histological grade (G3/Signet ring cell/ Mucinous vs. G1/G2) | 1.147(0.868-1.515) | 0.335 |  |  |  |
| Lymphovascular invasion (Yes vs. No) | 1.584(1.190-2.110) | 0.001 |  | 0.918(0.673-1.252) | 0.589 |
| Perineural invasion (Yes vs. No) | 2.214(1.666-2.941) | <0.001 |  | 1.496(1.089-2.055) | 0.013 |
| 5-Fu^a^ (No vs. Yes) | 1.436(1.088-1.896) | 0.010 |  | 1.648(1.235-2.198) | 0.001 |
| CD66b Non(high vs. low) | 1.018(0.778-1.331) | 0.896 |  |  |  |
| CD66b IM(high vs. low) | 2.090(1.589-2.749) | <0.001 |  | 2.055(1.407-3.000) | <0.001 |
| CD66b TC(high vs. low) | 1.588(1.212-2.080) | 0.001 |  | 0.827(0.570-1.200) | 0.317 |

Note: ^a^ Fluoropyrimidine-based adjuvant chemotherapy, mostly including capecitabine plus platinum, capecitabine alone, or S1 (combined tegafur, gimeracil, and oteracil), in patients at advanced stage or early stage tumors with lymph node metastasis in this retrospective study.

Abbreviations : DSS: disease special survival; Non: Nontumoral tissues; IM: invasive margin; TC: tumor center; HR: hazard ratio; CI: confidence interval; ASA: American Society of Anesthesiology.
