# Supplementary material for: Tumor-associated neutrophils induce EMT by IL-17a to promote migration and invasion in gastric cancer cells
Source: J Exp Clin Cancer Res. 2019 Jan 7;38:6. doi: 10.1186/s13046-018-1003-0 (PMC6323742; doi:10.1186/s13046-018-1003-0)
Supplement: Supplementary file 4 — Figure S1. Kaplan-Meier curves of DFS and DSS based on the number of IL-17a+cells in GC. (a, b) Higher number of 17a+cells in GC tissues were closely correlated with poor DFS and DSS (P < 0.001 and P < 0.001). (DOCX 144 kb) [file 13046_2018_1003_MOESM4_ESM.docx]

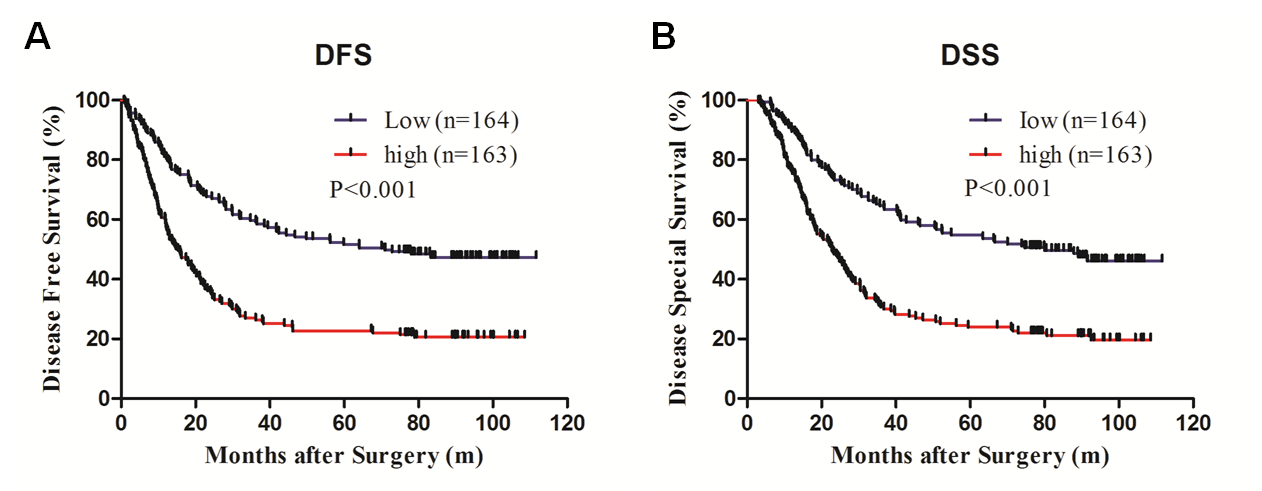


**Figure-1. Kaplan-Meier curves of DFS and DSS based on the number of IL-17a+cells in GC.** (a, b) Higher number of 17a+cells in GC tissues were closely correlated with poor DFS and DSS (*P* < 0.001 and *P* < 0.001).
